# Advancements in artificial intelligence applications for liver ultrasound imaging

**DOI:** 10.1093/bjrai/ubaf019

**Published:** 2025-12-17

**Authors:** Yu-Ting Shen, Hui-Xiong Xu, Bo-Yang Zhou

**Affiliations:** Department of Ultrasound, Zhongshan Hospital, Institute of Ultrasound in Medicine and Engineering, Fudan University, Shanghai 200032, China; Department of Ultrasound, Zhongshan Hospital, Institute of Ultrasound in Medicine and Engineering, Fudan University, Shanghai 200032, China; Department of Ultrasound, Zhongshan Hospital, Institute of Ultrasound in Medicine and Engineering, Fudan University, Shanghai 200032, China; Department of Medical Ultrasound, Center of Minimally Invasive Treatment for Tumor, Shanghai Tenth People’s Hospital, Ultrasound Education and Research Institute, School of Medicine, Tongji University, Shanghai Engineering Research Center of Ultrasound Diagnosis and Treatment, Shanghai 200072, China

**Keywords:** artificial intelligence, deep learning, machine learning, ultrasound, liver disease

## Abstract

Liver diseases consistently plague people’s daily lives as a result of their high morbidity and mortality rates. Ultrasound (US), favoured by its flexibility, free of radiation, cost-effectiveness, and real-time capabilities, has been commonly employed as one of the first-line imaging tools for hepatic conditions. Artificial intelligence (AI) algorithms are increasingly applied to automatically identify intricate patterns and perform quantitative analyses in US imaging, potentially reducing radiologists’ workload and improving diagnostic efficiency. AI-based US has been of substantial assistance in detecting, diagnosing, screening as well as monitoring of various liver diseases, and has attracted extensive attention among the medical community. In this review, we present a general introduction to AI in medical imaging; we next review its rapidly evolving applications in liver US, covering evaluation of hepatic steatosis severity, assessment of liver fibrosis, identification of focal hepatic lesions, preoperative prediction of high-risk pathological characteristics, assessment of postoperative prognosis, and the analysis of the model of integrated application of multi-omics data; finally, we present an outlook on the clinical applications of AI-based US in the liver diseases.

Key points

- AI algorithms are well suited for a US-inclusive field of medical imaging.
- Comprehensive understanding of AI-based liver US techniques to develop effective predictive models.
- AI will become a valuable tool in the field of liver US.

## Introduction

During the past few decades, liver diseases have plagued people and have relentlessly ascended to become a major contributor to death and illness globally. According to global cancer burden statistics, liver disease is responsible for approximately 2 million deaths throughout the world each year, of which liver cirrhosis ranks as the 11th cause of death and liver cancer as the fourth cause of death worldwide, together accounting for 3.5% of all global deaths.^1^^,^^2^ The most common reasons for cirrhosis include viral hepatitis, alcoholic as well as non-alcoholic fatty liver disease (NAFLD). Hepatitis B and C viruses can lead to viral hepatitis, cirrhosis, and notably, hepatophilic viruses represent an essential element in the majority of acute hepatitis cases, whereas drug-induced liver injury accounts for an increasing proportion of cases.^3^^,^^4^ An estimated 2 billion people worldwide consume alcohol with over 75 million diagnosed with an alcohol use disorder, and who are at risk of developing alcohol-related liver disease.^5^ Moreover, the increasing prevalence of obesity and diabetes contributes to the development of NAFLD and hepatocellular carcinoma (HCC). Approximately 2 billion adults are overweight or obese, and over 400 million have diabetes, both of which are significant risk determinants for liver diseases.^1^^,^^6^ Therefore, it is crucial to pay attention to liver disease, and early identification and screening are essential for tailored treatment of patients.

In clinical practice, ultrasound (US) serves as an indispensable tool for the detection, diagnosis, treatment, and monitoring of liver diseases owing to the advantages of being safe, non-invasive, cost-effective, real-time diagnostic features.^7^ US is recommended as the premier modality for the detection and diagnosis of liver disease by several guidelines, including the Asian Pacific Association for the Study of the Liver (APASL),^8^ World Federation for Ultrasound in Medicine and Biology (WFUMB),^9^ and European Federation of Societies for Ultrasound in Medicine and Biology (EFSUMB).^10^ Conventional US images provide valuable information about the morphology, texture, size, boundary, edges, and other acoustic features. Colour Doppler flow imaging (CDFI) reveals blood flow signals, while shear wave elastography (SWE) could evaluate tissue stiffness to enable US “palpation”. Additionally, contrast-enhanced ultrasonography (CEUS) obtains the results of the blood flow and perfusion patterns in the focal tissues, and the information from these images can help doctors to further diagnose liver diseases.^11–13^ However, the interpretation of liver US images depends quite on the subjective assessment and past experience of the US experts. Multimodal information on the same lesion is becoming increasingly available and data-rich, and accurate diagnosis of a lesion may require a background of knowledge in different fields, while the integration of multimodal data involves the exclusion of useless information, which is becoming highly demanding on the radiologists’ experience.^14^ Visual recognition and manual reading are not able to address this growth, and radiologists are faced with higher rates of missed and misdiagnosis due to disparities in expertise when dealing with large amounts of image data.^15^ The demand for effective management of enormous amounts of information has fuelled the immersion, evolution, and application of artificial intelligence (AI) and its related technologies in the medical field. AI-based US has significantly aided in the detection, diagnosis, and application of a wide range of liver diseases in recent years, drawing significant attention from the medical communities, especially with the assistance of machine learning (ML), deep learning (DL), and other derivative algorithms.^16^

The aim of this review is to provide a comprehensive overview of the rapid advancements in AI-driven ultrasound for liver disease management. Specifically, we seek to elucidate the core concepts of AI in medical imaging and then critically synthesize the growing body of evidence for its clinical applications in hepatology. This review will systematically cover the role of AI in the evaluation of hepatic steatosis risk stratification; assessment of chronic liver disease; estimation of hepatic fibrosis; segmentation, detection, and diagnosis of focal hepatic lesions; preoperative prediction of high-risk pathological characteristics; assessment of postoperative prognosis; analysis of the model of integrated application of multi-omics data. Finally, we discuss the challenges and outlook for the future of AI-based US in the clinical practice of liver disease.

## AI in medical imaging

**Figure 1.**
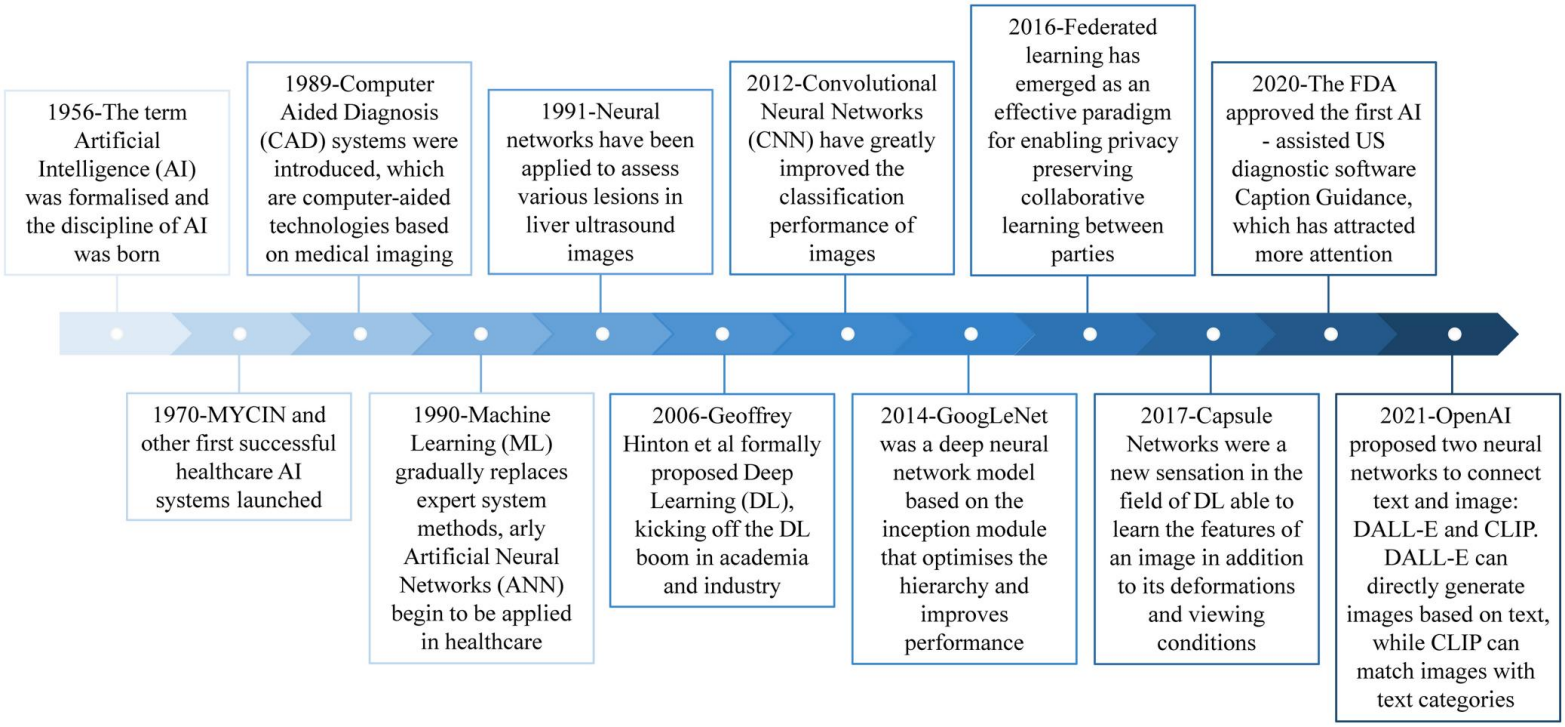
A diagram of the temporal development of the application of AI technologies. Abbreviations: AI = artificial intelligence; MYCIN = an early AI program.

The primary motivation for the emergence of AI in the field of medical imaging arises from the demand for improving the effectiveness and efficiency of clinical practice. Medical imaging data keeps growing at a rate that is disproportionate to the availability of trained radiologists, which has led to a dramatic escalation in the workload of doctors. Statistically, there are situations where radiologists have to interpret an image approximately every 3-4 seconds to meet their daily workload, forcing healthcare to develop new models for increasing productivity in order to compensate for the workload.^17–19^ AI technologies have been applied in several orientations in healthcare, such as tele-patient monitoring, robotic surgery, virtual assistance, as well as drug discovery and development.^20–23^ Particularly applicable in the areas of medical imaging, AI successfully identifies complicated imaging patterns derived from image data allowing better quantitative evaluation to be performed in an automated and reliable manner. The velocity at which AI is advancing in imaging is positively proportional to the exponential growth in data and computational capabilities, and the continued growth and development of AI has undergone several important journeys since the 1960s (Figure 1). The historical origins of AI can be traced back to 1956, when it was first formally introduced at a conference at Dartmouth.^24^ In 2006, Geoffrey Hinton and others formally proposed DL and started the DL craze in academia, which had a profound impact. Since 2012, the emergence of AI has been facilitated by the successful exploitation and deployment of image classifiers such as convolutional neural networks (CNNs).

**Figure 2.**
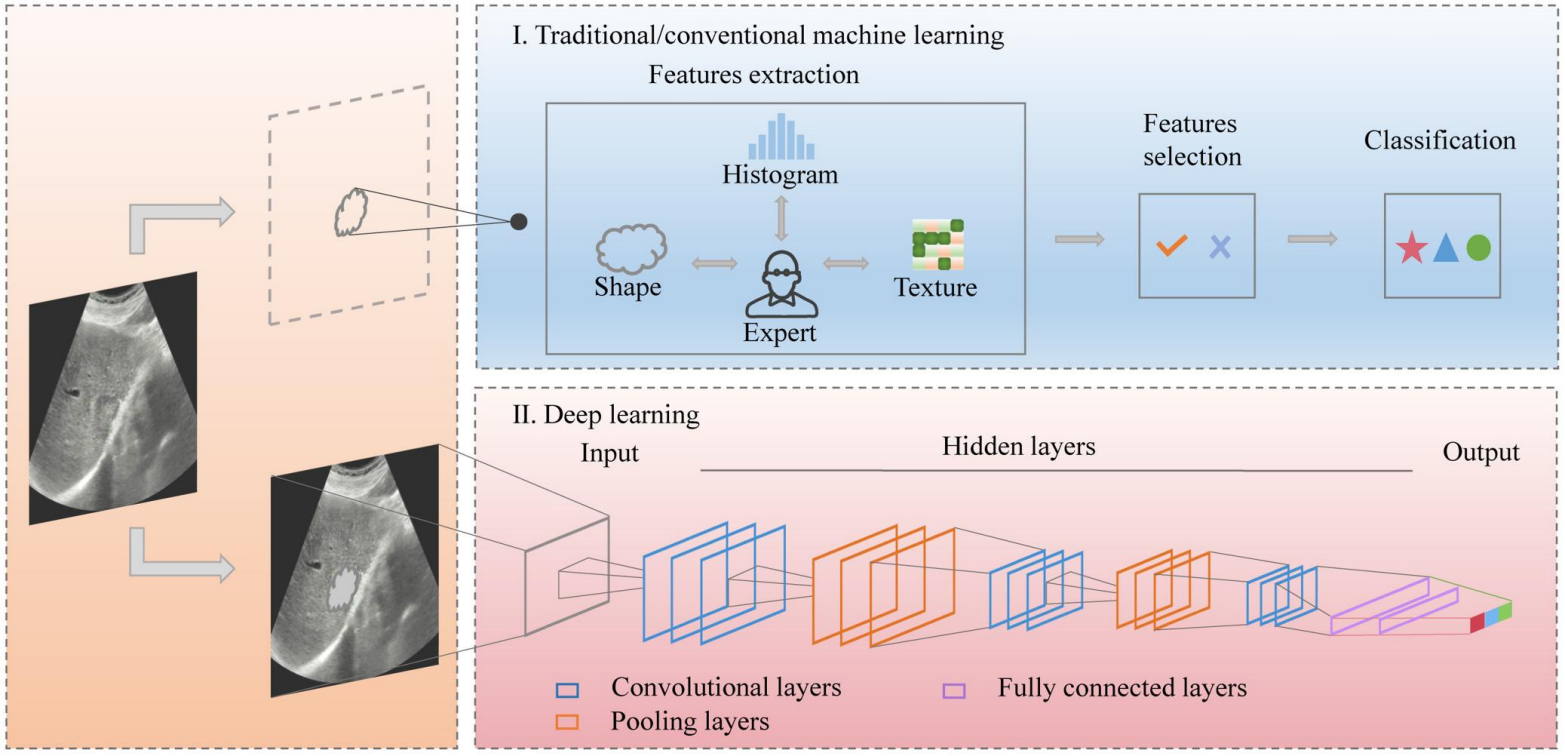
AI algorithms in medical imaging. There are 2 types of AI algorithms commonly employed currently, the upper section of the image representing traditional/conventional ML and the lower section of the image for the most up-to-date DL methods. Abbreviations: AI = artificial intelligence; DL = deep learning; ML = machine learning.

There are 2 types of widely used AI algorithms that we know of nowadays, namely traditional ML and the latest DL methods (Figure 2). ML is a subfield of AI, a term first introduced in 1959.^25^ Its primary feature is that manually extracted engineering features, such as texture, size, shape, areas of interest, as well as image pixel histograms, are used as inputs to ML, which can be trained to categorize images and output corresponding results.^26^ DL, a subfield of the ML algorithm, has received substantial attention in recent years. Its principal characteristic lies in its ability to automatically learn features within data, rather than the requirement of prior definition made by human specialists.^27^^,^^28^ DL, as a data-driven algorithm which cuts down on manual preprocessing steps to minimize inter-reader variability differences, is promising to efficiently improve the execution of clinical decisions. Notably, CNNs are currently the most widespread type of DL architecture in medical imaging.^29^ CNNs consist of a series of layers, including convolutional, pooling, fully connected as well as nonlinear layers, which progressively learn advanced imaging features.^30^ In addition, recursive neural networks are common algorithms for DL applications, featuring the ability to process image or numerical data, which can learn to have relevant data types due to the network’s inherent ability to memorize.^31^ Other DL algorithm applications are also applied to process medical imaging data sporadically, including recurrent neural networks, generative adversarial networks, autoencoders, transformers, and many other types.^32^^,^^33^

## Clinical application of AI-based US in liver diseases

**Figure 3.**
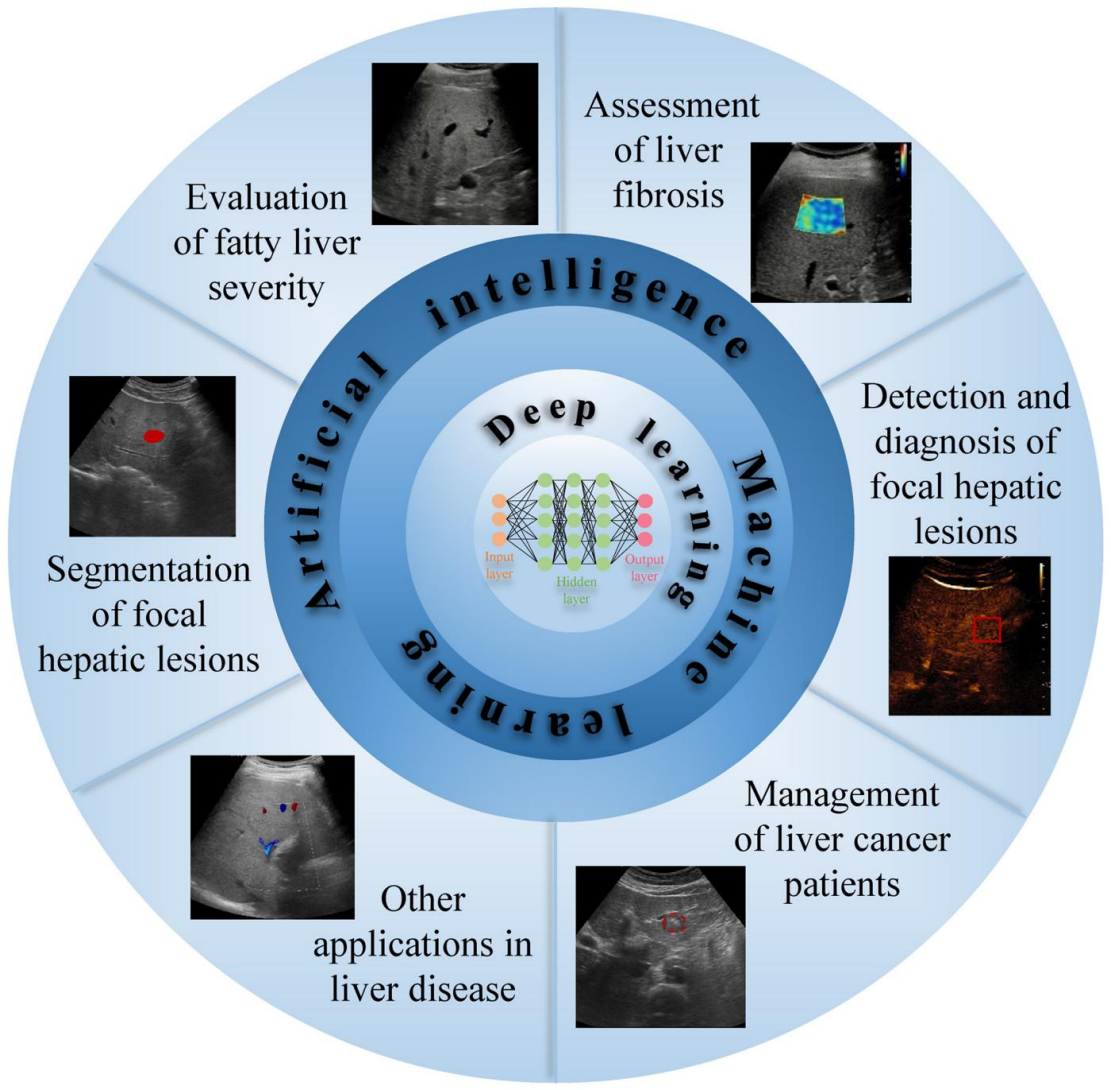
Clinical applications of AI-based US in liver diseases include risk stratification of fatty liver, assessment of chronic liver disease, segmentation, detection and diagnosis of focal liver lesions, management of patients with liver cancer, analysis of combined application models as well as adjunctive diagnosis of other diseases of the liver. Abbreviation: AI = artificial intelligence.

In terms of the imaging modalities involved, US is the most popular clinical technique for detecting liver disease owing to its affordability, portability, being free of radiation, safety, and availability of real—time scanning. Viewed from this point, AI-driven US provides greater advantages over CT and MRI as regards routine clinical applications.^14^^,^^34–36^ Consistently, radiologists could visually evaluate images to detect, describe, diagnose, as well as monitor liver disease, while US images vary depending on the operator and equipment. AI-assisted liver US diagnosis has become increasingly sophisticated, which is attributed to the advancement of US hardware as well as improvements in computing capabilities to recognize complicated imaging features in recent years. A comprehensive literature search was conducted, with electronic databases including PubMed, Web of Science, and Google Scholar systematically queried using a combination of keywords and Boolean operators. Core search terms included: (“artificial intelligence” OR “deep learning” OR “machine learning”) AND (“ultrasound” OR “sonography”) AND (“liver” OR “hepatic”) AND (“steatosis” OR “fibrosis” OR “focal lesion” OR “hepatocellular carcinoma”). The inclusion criteria prioritized original research articles, significant review articles, and consensus guidelines that focused on the development or clinical application of AI models in liver ultrasound (Figure S1). In the surveyed literature, clinical applications of AI-based US in liver diseases include risk stratification of hepatic steatosis, assessment of chronic liver disease, segmentation, detection and diagnosis of focal liver lesions (FLLs), management of patients with liver cancer, as well as analysis of combined application models (Figure 3).

### Identification or diagnosis of hepatic steatosis

The process in which lipid droplets appear or increase significantly in liver cells is described as steatosis that may result from metabolic syndrome, poor dietary habits, alcoholism, drug factors, obesity due to insulin resistance, and various other factors. With the progression of hepatic steatosis, it can subsequently develop into non-alcoholic steatohepatitis, followed by advanced liver fibrosis/cirrhosis and potentially HCC. It is imperative that patients with hepatic steatosis are accurately screened and graded for the treatment decisions that can be implemented to lessen the impact of the disease on the patient. The grading of hepatic steatosis severity by conventional 2D US mainly relies on the visual judgement of the observer, which includes evaluating dense parenchymal echoes, attenuation of far-field echoes, increased contrast between liver and kidney, as well as blurring of intrahepatic blood vessels, etc. However, the inter-observer agreement needs to be improved, highlighting the critical need for a quantitative and non-invasive method to assess the severity of hepatic steatosis disease. The application of AI-based US for risk stratification of hepatic steatosis is summarized in Table 1.

**Table 1.** Summarization of different applications of AI-based US in the assessment of hepatic steatosis and chronic liver fibrosis in the surveyed literature.

Ref.	Dataset size (*n*)	Algorism	Aim of the research	Performance
Kuppili et al (2017)^37^	16	Extreme Learning Machine (ELM)-based US system	Hepatic steatosis risk stratification	Accuracy: 96.75%
Biswas et al (2018)^38^	63	DL-based US paradigm	Liver detection and hepatic steatosis risk stratification	Accuracy: 99.00%
Saba et al (2016)^39^	62	Back-propagation neural network (BPNN)-based US paradigm	Hepatic steatosis classification	Accuracy: 97.58%
Che et al (2021)^40^	55	CNN-based US architecture	Hepatic steatosis risk stratification	Accuracy: 93.80%
Byra et al (2018)^41^	55	Inception-ResNet-v2 convolutional neural network-based US approach	Grading the degree of steatosis in the liver	Area under the ROC curve (AUC): 0.977
Chen et al (2020)^42^	205	US entropy imaging and VGG-16 model-based DL algorithm	Graded prediction of hepatic steatosis	The AUCs for grading mild, moderate, and severe hepatic steatosis were 0.710, 0.750, 0.880
Byra et al (2022)^43^	135	Transfer learning with CNN-based US model	Liver fat assessment	AUCs: 0.910 for proton density fat fraction (PDFF) ≥ 5% and 0.86 for PDFF ≥ 10%
Gao et al (2014)^44^	/	Multilayer feed-forward neural network-based US method	Classifier for five-stage prediction of hepatic fibrosis (S0-S4)	Accuracies of S0-S4 were 100%, 90%, 70%, 90%, and 100%, respectively
Kagadis et al (2020)^45^	200	DL-based shear wave elastography (SWE) model	Diagnosis and progress evaluation of chronic liver disease	AUC: 0.979-0.990
Gatos et al (2019)^46^	200	CNN-based SWE algorithm	Evaluate fibrosis staging	Accuracies of masked SWE images: 82.50%-95.50% and unmasked images: 79.50%-93.20%
Gatos et al (2017)^47^	126	Stiffness value clustering with ML-based SWE algorithm	Classifies chronic liver disease and healthy cases	Accuracy: 87.30% AUC: 0.870
Li et al (2019)^48^	144	ML-based multiparametric ultrasomics	Diagnostic for liver fibrosis stage	AUC: 0.850
Wang et al (2019)^49^	398	DL-based radiomics of elastography (DLRE)	Assess of hepatic fibrosis stages	AUC: 0.970 for cirrhosis, 0.980 for advanced fibrosis, 0.850 for significant fibrosis
Kutbuddin et al (2025)^50^	48	ML-based US architecture	Quantitative assessment of liver fibrosis	The consistency of fibrosis results exceeded 95%

Abbreviations: AI = artificial intelligence; CNN = convolutional neural network; DL = deep learning; ML = machine learning; ROC, = receiver operating characteristic curve; US = ultrasound.

Kuppili et al presented a strategy focusing on US Liver images characterized as normal or abnormal (hepatic steatosis afflicted) based on a reliable and speedy Extreme Learning Machine (one of the ML algorithms) using 3 methods (Gabor, Gray Level Run Length matrix, and Gray Level Co-occurrence Matrix) to extract 46 features for cross-validation of the output predictions.^37^ The results showed that the proposed model outperforms conventional ML methods in terms of specificity (97.59% vs 88.87%), sensitivity (94.23% vs 92.23%), accuracy (96.75% vs 89.01%) as well as area under the curve (0.97 vs 0.91). Biswas et al conducted a study that developed a DL-based US paradigm for liver detection and risk stratification for phenotypic characterization of hepatic steatosis, which was shown to achieve an accuracy of 99% superior to traditional ML approaches.^38^ Saba et al constructed a back-propagation neural network paradigm for hepatic steatosis classification with a high accuracy of 97.58%, achieving good application value.^39^ Ultrasound is the imaging modality of choice for detecting and classifying hepatic fatty infiltration by virtue of its wide availability and high cost-effectiveness. The hyperechoic nature of hepatic steatosis US images has been a challenge for detection and stratification performed based on traditional ML methods. Images based on the novel DL paradigm of US highlight characteristics of liver tissue (such as Gabor, GLCM, GRLM are based on the evaluation of texture features, scale and orientation of pixel distribution), providing essential image information to enhance the classification properties of the AI architecture. The above results revealed that the US model with novel ML/DL support improves the performance of hepatic steatosis risk assessment in terms of accuracy, precision, and assessment score of ML/DL. US-based AI improves the diagnostic performance for hepatic steatosis and has the potential to become an important diagnostic aid for radiologists. This demonstrates the potential of AI to provide a standardized, quantitative tool for steatosis grading, which could reduce inter-observer variability and support more consistent treatment decisions in the management of hepatic steatosis.

### Assessment of chronic liver fibrosis

Chronic liver disease is accompanied by changes in liver volume, contour, morphology as well as texture, which results in inflammation of the liver tissue leading to recurrent injury and fibrosis, known as the progression of excessive connective tissue. Correct differentiation of liver fibrosis advancement is useful in guiding the choice of clinical treatment regimen. SWE is an imaging technique that exploits changes in tissue elasticity induced by specific pathological/physiological conditions to assess tissue stiffness, and was first introduced in the 1990s.^51^ As this technique has evolved over the years, it allows for the quantitative estimation of tissue stiffness and is consequently of value in the assessment of excessive connective tissue progression in liver fibrosis. Recent studies have demonstrated the potential diagnostic value of the AI-assisted technique in comprehensively and objectively analysing liver US images (Table 1).

In a single-centre study by Gao et al employed Gray Level Gradient Co-Occurrence Matrix and Gray Level Co-occurrence Matrix (a texture extraction methodology) for texture analysis of 2D liver US images, and multilayer feed-forward neural network as a classifier for 5 stages prediction of hepatic fibrosis (S0-S4: graded from mild to severe hepatic fibrosis), and the prediction correctness rate of different classifications were all >70%.^44^ However, the grey scale detection method was greatly affected by different instruments and compensation for time gain. Another single-centre study by Kagadis et al suggested a DL train-and-test network using time-stable and complete SWE for the diagnosis and progress evaluation of chronic liver fibrosis.^45^ The model achieved superior diagnostic performance in terms of area under the operating characteristic curve (AUC) of 0.979-0.990 by using hepatic biopsy for reference, which far exceeded the results of radiologists (0.800-0.870). A prospective multicentre study developed DL-based radiomics of elastography with liver histology as a reference standard, which exhibited the best overall performance regarding the prediction of hepatic fibrosis staging when compared to 2D-SWE and biomarkers, with an AUC diagnostic of cirrhosis, advanced fibrosis and significant fibrosis were 0.97, 0.98, and 0.85, respectively.^49^ Gatos et al developed an algorithmic model of CNNs to automatically identify and isolate low-and high-stiffness time-stable regions in SWE images to evaluate fibrosis staging.^46^ The results showed that the accuracy of this approach in diagnosing fibrosis stage was higher in masked SWE images (82.5%-95.5%) than in unmasked images (79.5%-93.2%). An interesting study applied stiffness value clustering with an ML algorithm to differentiate between patients with chronic liver disease and healthy cases, which quantified the colour information of the stiffness results in the SWE images and classified the corresponding varying-coloured regions by associating them with a specific range of stiffness values.^47^

**Figure 4.**
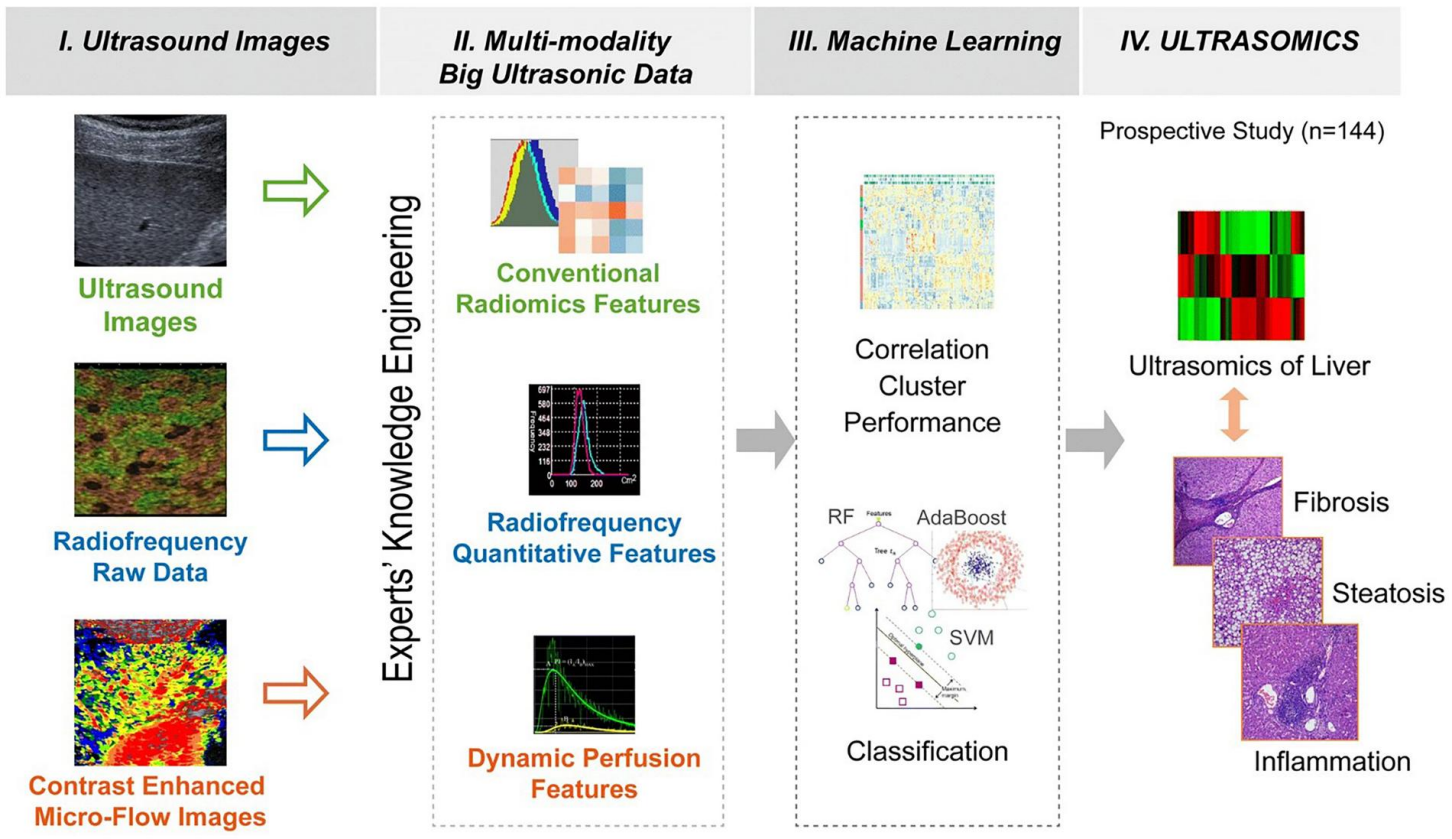
Flowchart of ML-based multiparametric US model for predicting liver fibrosis staging. (I) Multi-parameter US images from different US patterns. (II) Multi-parametric US images were extracted for each feature parameter. (III) ML was implemented to analyse the data in order to select the best predictors and test the prediction performance. (IV) Patients with liver fibrosis were recruited and an AI-based multiparametric US model was developed to predict liver fibrosis staging. Abbreviations: AI = artificial intelligence; ML, = machine learning; US = ultrasound. (Figure originally published in European Radiology by Li et al^48^).

In recent years, many studies have introduced SWE to associate liver stiffness with the fibrosis stage. Applying different types of ML/DL classical algorithms such as Naive Bayes, RF, KNN, SVM, GoogLeNet, AlexNet, ResNet50, DenseNet201, and VGG16, to construct decision-support systems based on real-time US hepatic tissue elastography for staging assessment of hepatic fibrosis. The non-invasive approach of assessment under the new AI algorithm provides an important diagnostic value for the different stages of liver fibrosis and even cirrhosis in patients with chronic liver disease (Figure 4).

### Segmentation, detection, and diagnosis of focal liver lesions

Focal liver lesions are defined as alterations in abnormal portions of the liver, primarily from hepatocytes, interparenchymal tissue and biliary epithelium, and US is the modality of first choice for their detection and diagnosis.^52^ Real-time segmentation of lesions is vital for further diagnosing and evaluating FLLs. There is not always an easy task for segmented liver images detecting FLLs (particularly in patients with cirrhosis) or characterizing the lesions encountered. Early AI-assisted diagnosis for distinguishing the nature of focal hepatic lesions in US images was mainly based on grey scale texture. The analysis was performed by extracting meaningful texture characteristics for the intra-and peripheral contrasts of the lesion, and with the advancement of fast, intelligent learning modes of DL neural networks, allowing for more robust assisted analyses of the nature of FLLs.

#### Segmentation of focal liver lesions

Segmentation studies in liver imaging exhibit significant specificity compared to other fields. There has always been a challenge in the segmentation of liver lesions; unlike other organs, the image contrast is relatively low between the background liver and the FLLs, which is a greater nuisance for the naked eye to recognize. With the introduction and application of DL segmentation techniques, it enabled a noticeable improvement in the segmentation accuracy, dice similarity coefficient of the liver tumour segmentation dataset that increased from 0.69 in 2018 to 0.867 in 2022.^53^ Typically, Ansari et al presented a new neural network architecture trained by a loss function, the Dense-PSP-UNet, which improves feature propagation while drastically reducing the number of parameters to achieve real-time and accurate segmentation of liver US images with Dice coefficients as high as 0.913^54^ (Table 2).

**Table 2.** Summarization of different applications of AI-based US in focal liver lesions in the surveyed literature.

Ref.	Dataset size (*n*)	Algorism	Aim of the research	Performance
Ansari et al (2023)^54^	8	Dense-PSP-UNet-based US model	Segmentation of liver US images	Dice coefficients: 0.913
Barash et al (2022)^55^	16	CNN-based intraoperative liver ultrasonography algorithm	Detection of focal liver lesions	Accuracy: 74.60%
Tosaki et al (2023)^56^	1996	YOLOv3-based US model	Detection of focal liver lesions	Detection accuracy was highest when the D/L ratio between 0.8 and 1.0
Ryu et al (2021)^57^	3873	CNN-based US model	Segmentation and classification of focal liver lesions	Accuracy: 82.20% AUC: 0.970
Xu et al (2023)^58^	11 468	LMC-Net-based US model	Segmentation and classification of focal liver lesions	Accuracy: 92.50% AUC: 0.983
Mitrea et al (2023)^59^	1441	CNNs and SAE-based US method	Diagnosis of hepatocellular carcinoma	Accuracy: 98.90% AUC: 0.997
Maclin et al (1992)^60^	64	Artificial neural networks trained on US data and laboratory test	Diagnose hepatic masses	Accuracy: 75.00%
Virmani et al (2013)^61^	56	Genetic algorithm-SVM-based US method	Characterization and classification of liver lesions	Accuracy: 88.80%
Acharya et al (2018)^62^	140	Computer-aided diagnosis (CAD) system	Diagnosis of focal liver lesions	Accuracy: 92.95%
Lassau et al (2019)^63^	46	AI-based US, CT and MRI system	Characterization of liver lesions	Accuracy> 90.00%
Schmauch et al (2019)^64^	544	DL-based US paradigm	Diagnosis of focal liver lesions	AUC: 0.891
Yang et al (2020)^65^	2143	Deep CNN-based US technique	Classification of focal liver lesions	Accuracy: 84.70% AUC: 0.924
Marya et al (2021)^66^	256	CNN-based EUS model	Differentiating focal liver lesions	AUC: 0.904
Xi et al (2021)^67^	596	DL-based US model	Classification of solid liver lesions	Accuracy in complete dataset: 0.840 accuracy in uncertain dataset: 0.790
Nishida et al (2022)^68^	29,264	Three CNN AI-based US model	Diagnosis of liver tumours	Accuracy in model-1, model-2, model-3: 80.00%, 81.80%, 89.10%
Ding et al (2025)^69^	3725	CEUS-based AI model	Multi-classification of FLLs	Accuracy: 85.00%-86.00%

Abbreviations: AI = artificial intelligence; AUC = area under the ROC curve; CNN = convolutional neural network; DL = deep learning; US = ultrasound.

#### Detection and diagnosis of focal liver lesions

**Figure 5.**
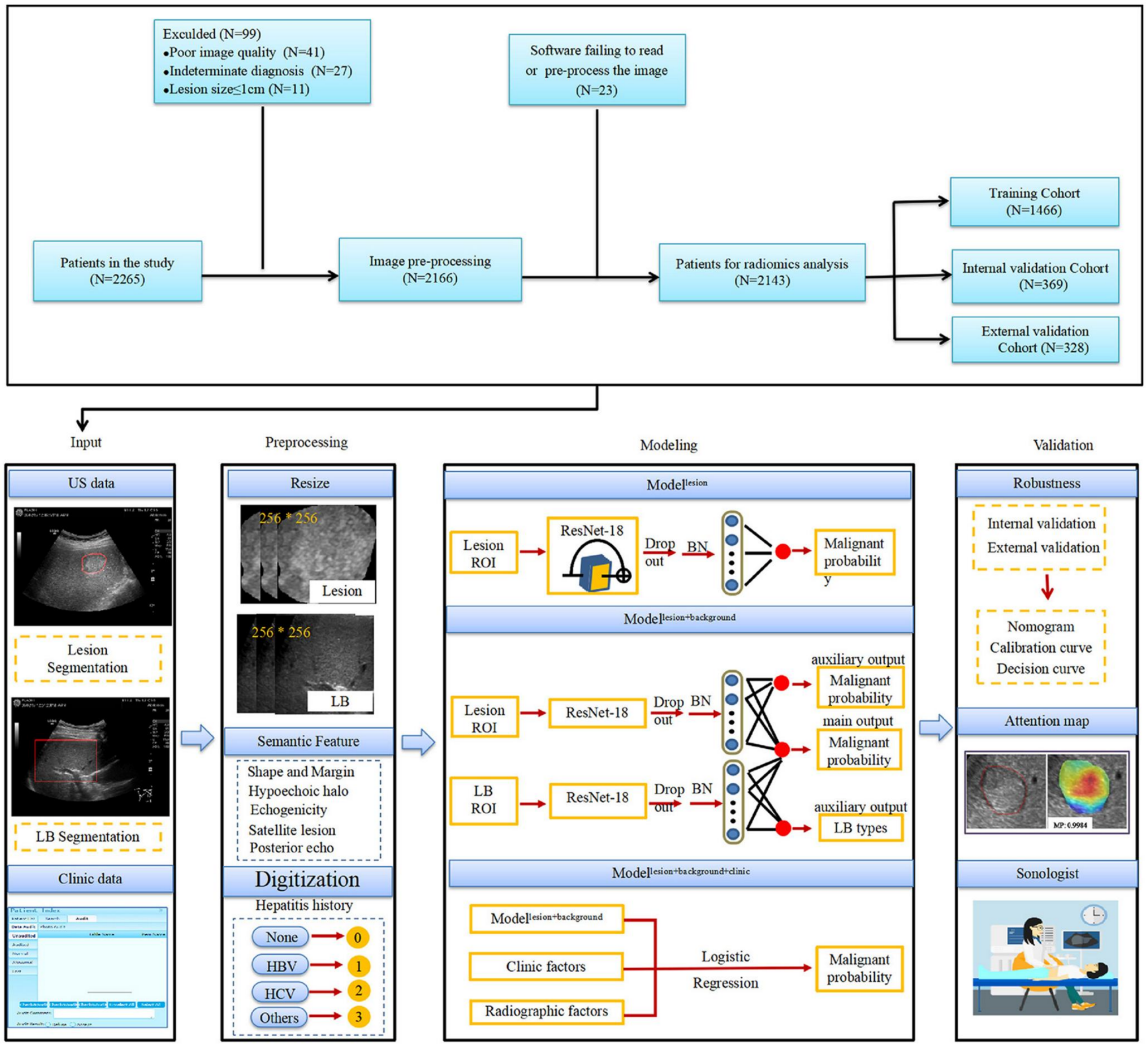
Flowchart of a deep CNN-based US algorithm for diagnosing the nature of focal liver lesions. Including image input, feature analysis, model development, testing and validation of data. Abbreviations: BN = batch normalization; CNN = convolutional neural network; HCV = heaptitis C virus; HBV = heaptitis B virus; LB = liver background; ROI = region of interest; US = ultrasound. (Figure originally published in EBioMedicine by Yang et al^65^).

The manifestations of most FLLs are intricate and atypical due to their pathological structure, in which these patients require invasive histological confirmation and/or close follow-up. Based on this condition, a variety of AI models have been developed to assist in improving the precision of non-invasive diagnosis of lesions. There is more literature on the application of AI-based US in the evaluation of FLLs, which we summarize in Tables 2 and 3. Firstly, in the application of accurate detection of FLLs: a single-centre study by Barash et al a dataset comprising 2576 cases labelled as normal liver tissue and 2467 cases labelled as containing FLLs was used to train and test a CNN for identifying cases of normal liver tissue and cases with FLLs in intraoperative liver ultrasound, revealing that this approach achieved a sensitivity of 99% and an accuracy of 74.6%.^55^ However, no external verification was conducted. In addition, in the study of Tosaki et al, they explored detection accuracy using multiple information regarding the area of the lesion and surrounding structures by varying the ratio of the maximum diameter of the liver lesion to the ROI size to build the YOLOv3 model for training and testing.^56^ The YOLOv3 model is a one-stage model which allows for the detection of targets in real time and at multiple scale. Secondly, applications in the diagnosis and classification of FLLs: Ryu et al and Xu et al developed CNN- and LMC (a specific network architecture name)-Net-based US models, which both integrated DL segmentation and classification practices, to diagnose the benign and malignant nature of FLLs in 400 and 735 US images, respectively, and the results demonstrated that the diagnostic accuracies of their proposed methods reached 82.2% and 92.5%.^57^^,^^58^ A valuable and multicentre study conducted in 13 hospitals, incorporating 24 343 US images, developed a deep CNNs-based US technique for benign and malignant classification of FLLs, which achieved a diagnostic AUC of 0.924 using liver biopsy and/or pathology results as a reference, with sensitivity and specificity better than that of a radiologist with 15 years of experience, and diagnostic accuracy equivalent to contrast-enhanced CT (both 84.7%) but slightly lower than contrast-enhanced MRI (87.9%)^65^ (Figure 5).

**Table 3.** Summarization of different applications of AI-based US in focal liver lesions in the surveyed literature.

Ref.	Dataset size (n)	Algorism	Aim of the research	Performance
Sato et al (2022)^70^	1080	Deep multimodal representation learning-based paradigm	Differentiation of liver tumours	Accuracy: 96.30% AUC: 0.994
Nakata et al (2023)^71^	6056	CNN-based US model	Classification of liver lesions	Accuracy: 78.30% AUC: 0.790
Brehar et al (2020)^72^	268	DL-based US method	Regional differentiation of hepatocellular carcinoma (HCC)	Accuracy for dataset GE7: 91.00%, AUC: 0.950 Accuracy for dataset GE9: 84.84%, AUC: 0.910
Sujana et al (1996)^73^	113	AI-based US method	Classification of liver lesions	Accuracy: 100.00%
Turco et al (2022)^74^	72	DL-based CEUS algorithms	Differential diagnosis of liver lesions	Accuracy: 84.00 %
Virmani et al (2014)^75^	108	Neural network ensemble-based CEUS algorithm	Differential diagnosis of liver lesions	Accuracy: 95.00 %
Sugimoto et al (2009)^76^	137	CAD-based CEUS system	Classification of focal liver lesions	Accuracy for metastasis, hemangioma and all HCCs: 84.80%, 93.30%, 98.60%
Gatos et al (2015)^77^	52	SVM-based CEUS algorithm	Classification of focal liver lesions	Accuracy: 90.30 %
Guo et al (2018)^78^	93	CAD-based CEUS system	Classification of liver tumours	Accuracy: 90.41%
Mitrea et al (2021)^79^	98	CNN-based CEUS and B-mode US method	Diagnosis of HCC	Accuracy: 97.00%
Zhang et al (2022)^80^	100	Mask region with CNNs-based multimodal US	Diagnosis of small liver cancer	Accuracy: 97.23% Average precision: 71.90%
Denis de Senneville et al (2020)^81^	46	CAD-based CEUS algorithm	Differentiate focal liver lesion types	Accuracy: 95.90% AUC: 0.970
Banerjee et al (2018)^82^	/	ML-based US LI-RADS algorithm	Classification of US LI-RADS categories	Average precision: 93.00%
Zhang et al (2021)^83^	153	Multi-kernel learning-based nonparallel hyperplane SVM-based CEUS algorithm	Diagnosis of liver cancer	Accuracy: 88.18%
Tangruangkiat et al (2024)^84^	581	ResNet50 (DL)-based US model	Classification of focal liver lesions	Accuracy: 87 ± 2.2%%
Chen et al (2024)^85^	465	DL-based CEUS algorithms	Differentiation of liver tumours	Accuracy: 86.00% AUC: 0.9237
Zhou et al (2024)^86^		CNN-based CEUS model	Malignancy diagnosis of liver lesions	AUC: 0.910
Kamiyama et al (2024)^87^	181	DL-based CEUS algorithms	Discrimination of liver lesions	Average correct answer rate: 60.1%; accuracy: 77.60%
Thodsawit et al (2023)^88^	260	CNN-based B-mode US method	Detection of liver lesions	FLL detection rate: 36.9, 667% (In experts)

Abbreviations: AI = artificial intelligence; AUC = area under the ROC curve; CAD = computer-aided diagnosis; CNN = convolutional neural network; CEUS = contrast-enhanced ultrasonography; DL = deep learning; LI-RADS = Liver Imaging Reporting and Data System; ML = machine learning; US = ultrasound.

In addition, imaging modalities exert a critical role in the assessment of FLLs; for instance, CDFI and CEUS have been conventionally used for the diagnosis of liver lesions.^89^ Compared to other enhancement techniques, CEUS enables every second of the examination to be viewed, recorded and traced without missing information and perfectly describes the contrast enhancement pattern of the liver lesion.^90^ Subsequently, in the application of AI-based CEUS liver lesion diagnosis: Turco et al and Virmani et al developed interpretable DL-based and neural network ensemble-based CEUS algorithms to assist radiologists in the differential diagnosis between benign and malignant liver lesions, respectively, which enable automated quantitative evaluation of the image information, reduce motion artefacts and the complexity of spatio-temporal nature of liver contrast enhancement in the image discrimination to achieve high classification accuracies of 84% and 95%, respectively.^74^^,^^75^ In addition, Denis de Senneville et al developed an algorithm—“Optical Flow”—to differentiate FLL types by simulating the human visual sensing of images for quantitative evaluation of the obvious microbubble transmission parameters evident on CEUS, which was demonstrated to achieve a high classification accuracy of 93.6%.^81^ It is well known that the American College of Radiology established the US Liver Imaging Reporting and Data System (US LI-RADS) in 2017 for standardizing terminology and protocol recommendations for the management of US examinations.^91^^,^^92^ The creation of a database of previous non-standardized reports and LI-RADS reports is a time-consuming and laborious challenge. Therefore, Banerjee et al proposed a scalable ML algorithm that can be applied to automate LI-RADS classification of large-scale US images, which can be data-mined and collected collectively to provide rationalized treatment for high-risk patients.^82^ Meanwhile, the US-based AI has also been utilized to differentiate different FLLs, including identifying benign from malignant lesions, and distinguishing different benign lesions of malignant lesions^76–79^ (Table 3).

In the field of analysis of FLLs, the utilization of AI to assist conventional US diagnostic techniques is promising. The precise AI-based segmentation of images (which means the recognition of regions of interest) is a prerequisite for implementing the intended detection or diagnostic task. Although the application of AI, especially DL, to liver lesions remains in the early exploratory phase, the results have confirmed that AI-based US achieves impressive performance in disease recognition, detection, as well as classification.

### Management of patients with liver cancer

**Figure 6.**
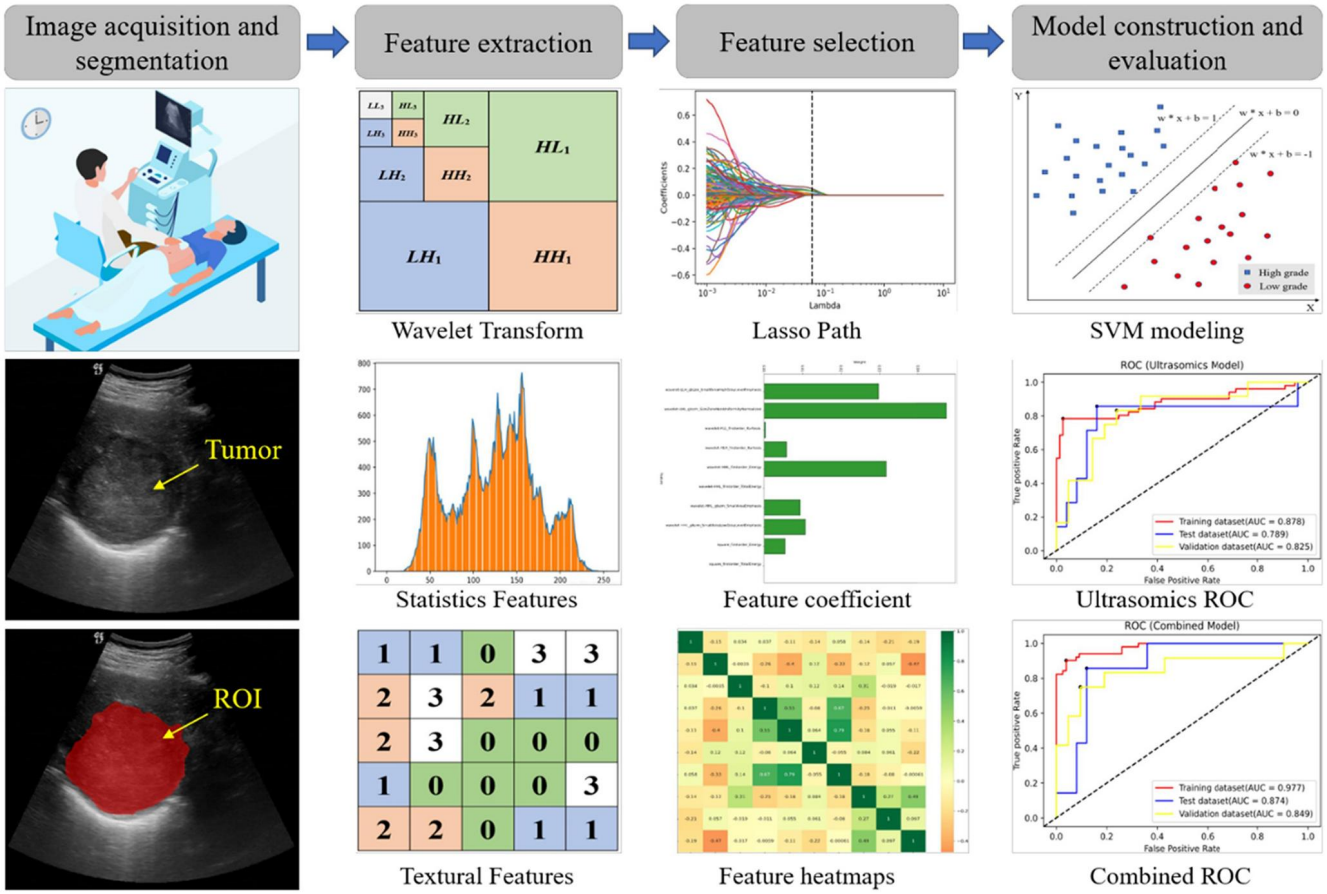
Flowchart of ML-based US model for prediction of preoperative pathological staging of HCC. Including image acquisition, feature analysis, and model development and assessment. Abbreviations: HCC = hepatocellular carcinoma; ML = machine learning; US = ultrasound. (Figure originally published in European Journal of Radiology by Ren et al^94^).

Liver cancer is the sixth most common cancer worldwide with the fourth leading common cause of cancer-related deaths.^93^ Advances in surgical, novel therapies, and systemic modalities for liver cancer have increased the sophistication in the management of patients. Based on this, accurate preoperative classification of cancer patients, assessment of cancer grading, analysis of treatment response, and prediction of postoperative recurrence, which enables flexible treatment allocation, is of great clinical significance for personalized patient management (Figure 6). AI-based US applications for the management of patients with liver cancer in the current studies include assessment of liver cancer classification and grading, prediction of microvascular invasion, evaluation of treatment efficacy of transarterial chemoembolization (TACE), and assessment of recurrence post-thermal ablation (Table 4).

**Table 4.** Summarization of different applications of AI-based US in the management of patients with liver cancer and other aspects of liver disease in the surveyed literature.

Ref.	Dataset size (*n*)	Algorism	Aim of the research	Performance
Hu et al (2019)^95^	482	US-based radiomics nomogram	Predicting microvascular invasion in HCC	AUC: 0.731
Zhang et al (2021)^96^	313	CEUS-based radiomics nomogram	Predicting microvascular invasion in HCC	AUC: 0.849
Mao et al (2021)^97^	114	ML-based US algorithm	Differentiate between primary and metastatic liver cancer	Accuracy: 84.30% AUC: 0.816
Ren et al (2021)^94^	193	SVM-based ultrasonomics model	Prediction of pathological grading of HCC	Accuracy: 81.82% AUC: 0.849
Liu et al (2020)^98^	419	DL-based radiomics model	Prediction of progression—free survival with RFA and SR	C-index: 0.726 for RFA, 0.741 for SR
Liu et al (2020)^99^	130	DL-based radiomics model	Prediction of personalized response of HCC to the first TACE treatment	AUC: 0.930
Bai et al (2021)^100^	60	Novel AI-based CEUS method	Evaluate postoperative recurrence treated with RFA	Detection rate: 92.30%
Zhao et al (2023)^101^	207	DL-based CEUS method	Evaluate postoperative recurrence treated with RFA	AUC: 0.760
Yang et al (2023)^102^	6784	Deep CNN-based US model	Identification of hepatic echinococcosis	AUC: 0.942 [0.904-0.967]
Wu et al (2022)^103^	967	Deep CNN-based US model	Classification of hepatic echinococcosis	Accuracy: 90.60%
Cheng et al (2022)^104^	5028	CAD-based US system	Classification of hepatic echinococcosis	Accuracy: 86.20%
Ma et al (2021)^105^	318	Dynamic CEUS radiomics model	Prediction of recurrence after thermal ablation	AUC: 0.840 C-index: 0.770
Zhang et al (2020)^106^	82	CNNs-based US method	Evaluating thermal lesions elicited by thermal ablation	AUC: 0.895
Yagasaki et al (2020)^107^	/	DL-based US system	Estimating liver motion in 3 dimensions	Accuracy: 97.50% AUC: 0.984
Xu et al (2010)^108^	/	Robotic assist system	Precision thermal ablation of liver tumours	Accuracy: 1.8 ± 0.9 mm
Schlosser et al (2016)^109^	/	Robotic assist system	Precision thermal ablation of liver tumours	Guidance rate: 80.00%
Daunizeau et al (2020)^110^	/	Robotic assist system	Precision thermal ablation of liver tumours	Relative error volume estimation: 4 ± 5%
Chen et al (2025)^111^	1030	A generative adversarial network-based model was build for direct pseudo-CEUS generation from B-mode US	Evaluate the effect of tumour ablation	SSIM values ranged from 0.84 ± 0.08 to 0.88 ± 0.04

Abbreviations: AI = artificial intelligence; AUC = area under the ROC curve; CAD = computer-aided diagnosis; CEUS = contrast-enhanced ultrasonography; CNN = convolutional neural network; DL = deep learning; HCC = hepatocellular carcinoma; ML = machine learning; RFA = radiofrequency ablation; SR = surgical resection; SSIM = structural similarity index; TACE = transarterial chemoembolization; US = ultrasound.

Hu et al developed a US-based radiomics nomogram for predicting microvascular invasion in liver cancer preoperatively, which is superior to the clinical nomogram for detecting the manifestation of microvascular invasion (AUC: 0.731 vs 0.634).^95^ Mao et al applied the 5 ML-based US algorithms and extracted 7 types of US image features to preoperatively and non-invasively differentiate between primary and metastatic liver cancer, and concluded that the logistic regression algorithm was observed to have achieved the best classification results with an accuracy of 84.3%.^97^ Ren et al proposed a combined SVM-based ultrasonomics model for preoperative prediction of pathological high-grade and low-grade grading of HCC, and the results showed that the best AUC of the combined model’s prediction reached 0.849, higher than that of the clinical model (0.770) and the ultrasonomics model (0.825).^94^ Liu et al developed DL-based radiomics models for the prediction of progression-free survival with surgical resection and radiofrequency ablation as well as personalized response of HCC to the first TACE treatment, respectively, with reference to the actual outcome of each patient, and the results of the researches demonstrated that the model enables personalized prediction in the preoperative period, which helps to optimize the patient’s curative choices.^98^^,^^99^ In 2 enlightening studies, Bai et al and Zhao et al developed a novel AI-based CEUS method and DL-based CEUS model to evaluate postoperative recurrence in patients with liver cancer/colorectal tumour liver metastasis treated *via* radiofrequency ablation.^100^^,^^101^ The former compared it with MRI and contrast-enhanced CT (CE-CT) examination approaches. The results indicated that the detection rate of the novel AI-based CEUS method at 2-year follow-up was 92.3% similar to that of CE-CT (96.2%) and higher than that of MRI (46.2%).^100^ The latter model was observed to have the superior predictive performance of the DL model to the clinical model at 56 months of follow-up observation (AUC: 0.76 vs 0.67).^101^ According to reports, the US, especially CEUS, is of great value in assessing the efficacy of targeted combined immunotherapy in patients with advanced HCC and the efficacy of chemotherapy combined with targeted therapy in patients with colorectal liver metastases.^112^^,^^113^ However, the application of AI-based US for patients with liver tumours in this area has not been tapped, which can be an important direction for subsequent research. Preoperative precision assessment of liver cancer patients and development of AI-based US clinical decision-support systems would be able to help physicians choose better therapeutics for their patients.

### Other applications in liver disease

Other applications based on AI-US in liver diseases include the following: for instance, identification of different subtypes of hepatic echinococcosis,^102–104^ assessment of liver thermal injury,^106^^,^^108^ prediction of liver 3D motion,^107^ robot-assisted guidance systems,^109^^,^^110^ etc. (Table 4). A significant study, involving 87 hospitals in China, has developed the first deep CNN model for differential diagnosis of hepatic echinococcosis based on US images.^102^ In this study, a total of 9631 liver US images were collected from January 2002 to December 2021 for model training and testing, and the developed deep CNN model was able to accurately discriminate hepatic echinococcosis from other FLLs, with diagnostic efficacy exceeding that of senior sonographers in endemic areas of hepatic echinococcosis.^102^ Zhang et al proposed a CNN-based US method for evaluating thermal lesions elicited by thermal ablation in porcine livers, in which features associated with thermal lesions were acquired by backscattering the thermal ablation region in the image, and ultimately achieving a satisfactory predictive performance (AUC = 0.895).^106^ An interesting research has developed that a DL-based US system available for estimating liver motion in 3 dimensions serves as an essential method for tumour tracking, which can considerably improve the problem of lost field of view for tumour tracking in 2D images.^107^ Currently, robotic assistance systems serve as a useful tool in surgical precision operations. Several studies have focused on the current prevalence of US-guided high-intensity focused US ablation treatments, where accurate image guidance and precise needle placement could potentially increase the efficacy of the treatment. Therefore, they proposed the application of a robotic assist system (combined imaging, needle actuation, and real-time navigation and positioning) to decrease uncertainty-induced interference and specifically improve the therapeutic effectiveness of thermal ablation.^108–110^

### Combined application model to assess liver disease

It is undisputed that liver diseases are complex and diverse, while single diagnostic models lack specificity and have limited assessment efficacy. With the continuous expansion of imaging and clinical data, the multimodal data integration of joint diagnostic models has been increasingly popular in clinical practice, as it improves diagnostic efficacy while increasing the reproducibility of the operation.^114^ AI-supported US-based joint diagnostic modelling includes combination with other imaging data (CT and MRI), integration with serologic indicators, and so on. Wei et al proposed a novel DL-based US, CT/MRI fusion pipeline, which employed a classification network to estimate the angle of the US probe, and after evaluation, formulated the problem in terms of a segmentation task to estimate the US plane within the 3D CT/MR *via* segmentation to align the interface from the 2D US image to the 3D CT/MR image, providing precise and clear image guidance for the ablation of liver tumours.^115^ In addition, a provocative research by Procopet et al achieved excellent diagnostic performance with >80% accuracy in patients with cirrhosis, portal hypertension, and/or oesophagal varices by introducing transient SWE based on artificial neural networks combined with 6 serologic scores (aspartate aminotransferase, alanine aminotransferase, international normalized ratio (INR), platelet count, albumin, and bilirubin).^116^ It is noteworthy that a new non-invasive diagnostic strategy termed “liquid biopsy” is a highly promising diagnostic modality for liver lesions and even early HCC, in which the key components, circulating tumour cells as well as circulating tumour DNA detection, have already yielded encouraging results. However, the combined application of AI-based US imaging technology and liquid biopsy has not been studied accordingly, which deserves further in-depth exploration.

## Discussion and outlook

AI algorithms, especially DL, have made great strides in the tasks of image recognition. Applications of various methods from traditional ML to CNNs to variational autoencoders have flourished in the field of medical image analysis, which has propelled its rapid advancement. The development of AI models that utilize US images has been accelerated over recent years, where they could support physicians in providing more accurate and efficient diagnosis, thus alleviating the burden on radiologists. With the availability of high-end US devices, images derived from SWE and CEUS, apart from greyscale images, are emerging as potential datasets for AI diagnostics in liver disease analysis.^117^ AI-based US patterns have been demonstrated to be beneficial in the assessment of diffuse liver disease, focal hepatic lesions, and other conditions. For instance, AI models have shown effectiveness in evaluating the severity of hepatic steatosis diseases and the degree of liver fibrosis, segmenting and classifying benign and malignant lesions in the liver. Additionally, these models have been explored for applications in determining the efficacy of TACE, predicting microvascular invasion (MVI) invasion in HCC, as well as the outcomes following thermal ablation. The implementation of AI-supported multi-omics modelling of liver US images combined with other imaging techniques and clinical data exerts a profound effect on the diagnosis and treatment of liver diseases. AI programs operating in a single mode that ignore the broader clinical context inevitably reduce their application potential. With the tracking and advancement of radiology, histopathology, genomics, and clinical information, integrating and analysing multimodal structured data provides new opportunities to surpass the diagnostic performance of traditional models. The clinical value of AI, framing the presented results not just as technical achievements but as steps toward addressing tangible clinical problems like reducing diagnostic delays, personalizing treatment plans, and alleviating doctor workload.

It has been recognized that AI-assisted liver US intelligent diagnostic performance, precision and efficiency have been improved accordingly. Consequently, the product transformation of AI research results is also gradually reflected in people’s eyes. In 2020, the FDA approved the first AI-assisted US diagnostic software Caption Guidance. The AI-US field has ushered in a major breakthrough, which has attracted more attention.^84^ In addition, GE Healthcare launched the wireless handheld Vscan Air US device (Wisconsin, United States), Clarius Mobile Health released the Clarius HD3 handheld US product (Vancouver, Canada), Demetics Medical Technology developed the AI-SONIC series products (Zhejiang, China), CHISON designed the Sono AI software (Jiangsu, China), and FYNNO launched the VINNO 10P portable colour US device (Jiangsu, China), and so on, a variety of US AI products continue to improve the performance of liver US in clinical practice.

The application of AI in the field of liver US imaging research has yielded promising results, although several limitations have been noted. Firstly, the comprehensiveness of the coverage of the dataset needs to be refined, with limited availability of completely labelled datasets and the requirement for rigorous ethical regulations. Secondly, the recognized “black box” nature of AI algorithms, especially DL algorithms, and the interpretability of decisions/predictions produced by these algorithms, demand further exploration.^36^ With the formal introduction of AI technology and the deployment of logical and statistical model recognition in medicine back in the early 1960s, many clinical tasks have been automated for identification and quantitative computation facilitated by AI. However, the theoretical understanding of the results of DL and CNN model export remains to be explored-which underlies why the multiple layers located between the input and output are referred to as “hidden layers” (Figure 1, II). Absence of transparency in modelling algorithms makes it challenging to predict the source of failure (hardware devices, software facilities or individual patient differences) and to isolate the mechanics of particular conclusions. Therefore, predictive modelling based on interpretable AI (XAI) algorithms-principle analysis of input images-is an important breakthrough bottleneck for future research. Third, most of the surveyed studies on AI-applied liver US imaging were retrospective and trained based on limited data obtained from a single hospital, which may involve data selection bias and an insufficient amount of data in the training set. Therefore, future research requires the need for large-scale prospective trials to generate Level 1 evidence, the importance of standardized benchmarking for generalizability, and the development of explainable AI (XAI) to build clinical trust. Furthermore, we highlight the transformative potential of integrating AI-analysed ultrasound features with multi-omics data for personalized medicine and stress the necessity of workflow and cost-effectiveness studies to demonstrate tangible clinical impact.

Extensive screening of liver US images requires considerable endeavor, which is both time-consuming and potentially prone to human error. In this context, AI becomes an invaluable tool for clinicians. It is important to be aware that integrating AI with clinical practice involves a transformation of the traditional roles of healthcare professionals along with ongoing training and upgrading to guarantee that AI is employed in an effective and ethical manner. By critically evaluating and integrating AI as a powerful decision-support tool into routine practice, clinicians can harness its capabilities to enhance diagnostic confidence, standardize assessments, and ultimately pave the way for more personalized and effective patient management strategies in hepatology. The full potential of AI in liver ultrasound can be realized through sustained efforts in robust validation, the development of explainable systems, and, crucially, through close collaboration among AI researchers, sonographers, hepatologists, and radiologists.

## Supplementary Material

ubaf019_Supplementary_Data
