# Supplementary material for: Advancements in artificial intelligence applications for liver ultrasound imaging
Source: BJR Artif Intell. 2025 Dec 17;3(1):ubaf019. doi: 10.1093/bjrai/ubaf019 (PMC13222679; doi:10.1093/bjrai/ubaf019)

**Advancements in Artificial Intelligence Applications for Liver Ultrasound Imaging**

**Figure S1**. Flowchart of the literature search and selection process.


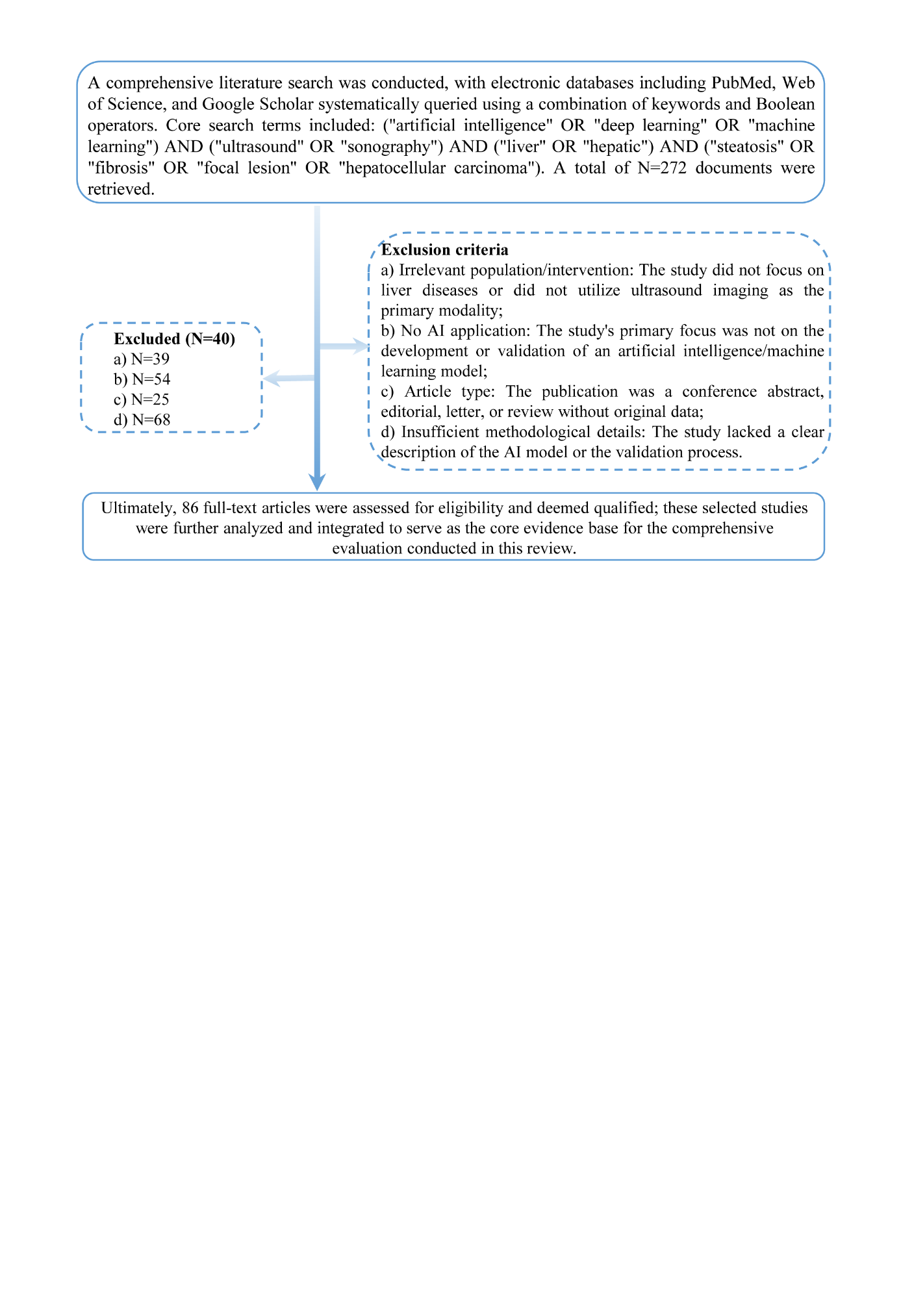

Supplement: ubaf019_Supplementary_Data [file ubaf019_supplementary_data.zip › Supplemental Material.docx]
