# Supplementary figures and images for: Advancements in artificial intelligence applications for liver ultrasound imaging
Source: BJR Artif Intell. 2025 Dec 17;3(1):ubaf019. doi: 10.1093/bjrai/ubaf019 (PMC13222679; doi:10.1093/bjrai/ubaf019)

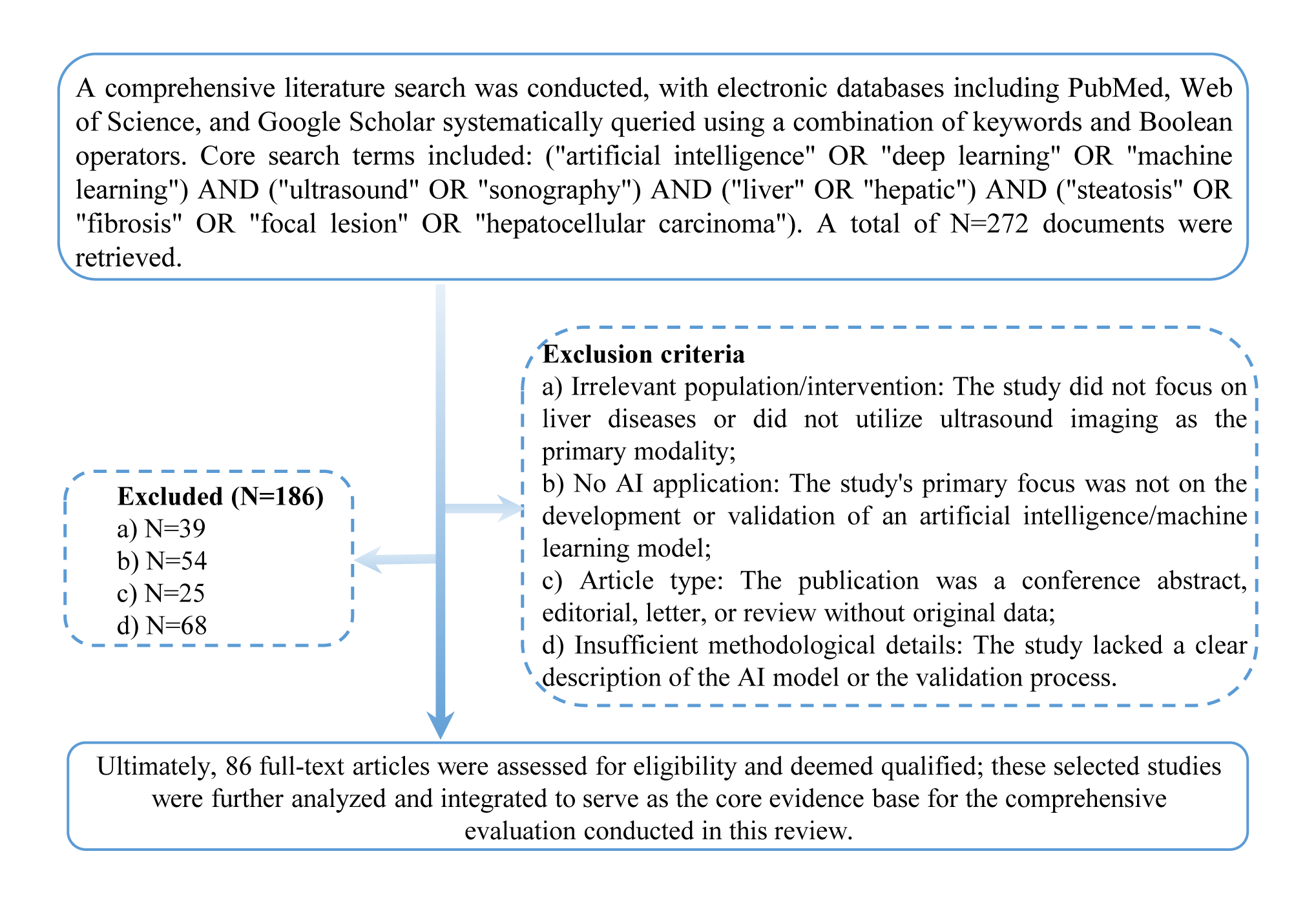

Supplement: ubaf019_Supplementary_Data [file ubaf019_supplementary_data.zip › Figure S1 (002).tif]
